# Removal of Heavy Metals from Mine Tailings in Central Chile Using *Solidago chilensis* Meyen, *Haplopappus foliosus* DC, and *Lycium chilense* Miers ex Bertero

**DOI:** 10.3390/ijerph20032749

**Published:** 2023-02-03

**Authors:** Pamela Lazo, Andrea Lazo, Henrik K. Hansen, Rodrigo Ortiz-Soto, Marcela E. Hansen, Felipe Arévalo, Claudia Gutiérrez

**Affiliations:** 1 Instituto de Química y Bioquímica, Facultad de Ciencias, Universidad de Valparaíso, Avenida Gran Bretaña 1111, Playa Ancha, Valparaíso 2360102, Chile; 2 Departamento de Ingeniería Química y Ambiental, Universidad Técnica Federico Santa María, Avenida España 1680, Valparaíso 2390123, Chile; 3 Escuela de Ingeniería Química, Pontificia Universidad Católica de Valparaíso, Avenida Brasil 2162, Valparaíso 2340025, Chile

**Keywords:** phytoremediation, heavy metals, removal

## Abstract

Mining activities have been a part of the history of Chile since time immemorial, generating pollution and environmental liabilities. Due to the lack of regulation, many tailings are deposited close to rivers or/and on unstable ground, near which towns have been built, generally in locations with no budget for their treatment. This study tested three plant species from Northern and Central Chile to remove total chromium, nickel, and zinc from tailings: *Solidago chilensis*, *Haplopappus foliosus,* and *Lycium chilense*, which complements the few existing studies on heavy metals removal with native or endemic Chilean shrubs. The experiments were conducted ex situ, and the initial and final concentrations of metals were determined in tailings and plants to obtain the removal efficiency, translocation and bioconcentration factors. Among these species, the best performance was obtained using *Solidago chilensis*, achieving removal efficiencies of 24% for Cr, 19% for Ni, and 17% for Zn, showing the ability to phytostabilize chromium and the higher resistance concerning the toxicity threshold. *Haplopappus foliosus* and *Lycium chilense* presented a slight tendency to stabilize chromium. Only *Solidago chilensis* showed little ability to extract Zn.

## 1. Introduction

Copper mining is one of the main economic activities in Chile. Chilean Legislation (Supreme Decree N°248/2007) establishes the concept of tailings as a finely ground solid that is discarded during mining operations and is not considered hazardous waste [1], but the lack of regulations has for many years caused many residues to be stored without environmental considerations. In 2004, Supreme Decree N°132 modified the Mining Safety Regulations by incorporating rules on the closure of mining operations, forcing, for the first time, the execution of a closure plan by mining companies at the end of their production phase, including the closure of tailings impoundments; however, the old mining operations not covered by this regulation generated a considerable number of abandoned tailings [1,2]. In addition to the above, Chile lacks restrictions on maximum concentrations of heavy metals in soils in the context of other environmental problems, and depending on the similarity of environmental conditions, the laws of foreign countries are used [3].

Physical stability and environmental impacts are among the most significant environmental and health concerns associated with the contamination by mining activities and environmental liabilities like abandoned tailings [4]. These environmental impacts include soil pollution by heavy metals, filtration of contaminated water into the environment and infiltration into the underground layers, pollution of the soil and air due to lifting and dragging of fine material, and contamination of superficial water bodies and plants, even in areas far from impoundments [5,6,7,8,9,10,11]. Moreover, several studies have evidenced the vulnerability of people living near polluted mine areas [12,13,14,15,16].

Three of the most critical environmental pollutants relating to mining activities are chromium, nickel, and zinc [4,17], where elements such as Cr and Ni could cause effects human health depending on the type of exposure, and mining sites are the primary sources [18].

Some heavy metals like Cr are not essential for living organisms; this element presents extreme toxicity even at trace levels, and has been recognized as one of the greatest dangers to all life forms, including humans [19,20]. Its mobility in soil depends upon the species. It is commonly found in the trivalent state in nature, which has very low solubility and reactivity; a low pH also facilitates chromium’s leaching in the soil [21]. The World Health Organization (WHO) indicates that the average concentration of total Cr in soils is between 14 and 70 mg·kg^−1^ [22]. The toxicity of chromium in most plants occurs at a concentration, as total chromium, of 5 mg kg^−1^ [23].

The concentration of Zn in plants is typically between 30 and 100 mg·kg^−1^ dry matter, the required concentration of Zn for crops is in the range of 15 to 20 mg·kg^−1^ dry weight [24,25], and more than 300 mg·kg^−1^ is toxic for most plants [26]. The mean concentration of Zn in the soil is 64 mg·kg^−1^ dry weight [26]. Nickel is an essential element for plants at concentrations between 0.05 and 10 mg·kg^−1^ dry weight; at higher values, it is toxic for plants [27].

Heavy metals participate in a series of reactions, like ion exchange, precipitation, adsorption, and complexation; therefore, their forms and availability change with time [28,29]. Among the techniques used to remediate soils with heavy metals include physical, chemical, and biological methods [29], phytoremediation corresponds to the latter.

Several studies in phytoremediation of tailings have been conducted using non-native species. However, an essential factor for successful processing is establishing an effective plant community; therefore, promising research in the area has been carried out with native species [30].

Chaabani et al., 2017, studied the removal of Pb and Zn from abandoned tailings in Tunisia with native species collected in situ; they showed a high tolerance to Pb, Zn, and Cd and a phytostabilizer character [31]. The study of Murtić et al., 2021, analyzed native plants such as *Urtica dioica* L., *Artemisia vulgaris* L., *Mentha arvensis* L., *Trifolium repens* L., *Medicago sativa* L., *Urtica urens* L. and *Achillea millefolium* L. in heavy-metal-polluted soils in Bosnia and Herzegovina, none of which showed a hyperaccumulator character for Ni Cr, Cd or Pb; in some of them, very high concentrations—above the toxicity threshold—of these elements were detected [32]. Additionally, Chandra et al., 2018, determined the concentration of several heavy metals in plants growing naturally in India; *Saccharum munja*, *Cynodon dactylon*, *Pennisetum purpureum*, *Rumex dentatus,* and *Basella alba* exhibited potential for phytoextraction of Zn, Cu, and Ni. Most plants had a high capability of heavy metal accumulation, mainly for Fe and Cu, where higher concentrations were detected in roots [33].

In Chile, previous studies in phytoremediation with endemic or native flora have been performed. For example, Lam et al., 2017 studied the native tree *Prosopis tamarugo* for tailings remediation, comparing the process with the tailings without amendments and using two treatments, one with CaCO_3_ and compost and a second with CaCO_3_ and arbuscular mycorrhizal fungi. The translocation factor was between 1 and 1.5 in the tailings without treatment for Zn and Cd, and the bioconcentration factor was higher than one for Cd with the second treatment; the comparison was carried out with introduced species, and less promising results were obtained with native species [34]. Milla-Moreno et al., 2020, studied the use of *Schinus polygama* (Huingán), *Baccharis linearis* (Romero), *Trevoa quinquenervia* (Tralhuén), and *Muehlenbeckia hastulata* (Quilo), all native Chilean evergreen shrubs, higher BCF and TF were determined for Ni, Zn, Cr among other metals, with Tralhuén [35].

In Chile, there are several tailings impoundments close to population centers like in Copiapó and Salamanca, among others. Considering the reduced budget of local governments in charge of these environmental liabilities, which at many times put the population at risk, the focus of the present study is on the evaluation of the removal of Cr, Ni, and Zn from mine tailings by endemic or native species from Northern and Central Chile, like *Solidago chilensis* Meyen, *Haplopappus foliosus* DC, and *Lycium chilense* Miers ex Bertero var. *chilense*, with low water requirement and good resistance.

This approach allows the implementation of an economical and environment-friendly method to decrease the mobility of these pollutants, the inherent risk of leaching, and the subsequent contamination of subsoil or groundwaters.

## 2. Materials and Methods

### 2.1. Chilean Bushes

Three species of bush were used: *Solidago chilensis*, *Haplopappus foliosus,* and *Lycium chilense*; all species grow in the Northern and Central Zone of Chile. The selected species have ornamental value and no frost resistance. *Solidago chilensis*, commonly called Fulel, is a frequently occurring native species belonging to the *Asteraceae* family. *Haplopappus foliosus* belongs to the *Asteraceae* family, a common endemic species called Cuerno de cabra. Finally, *Lycium chilense* is a rare native species belonging to the *Solanaceae* family with the common name of Coralillo [36].

### 2.2. Quality Control and Assurance

Inductively coupled plasma–optical emission spectroscopy (ICP-OES) was used to determine the elemental composition. The equipment used was a Perkin Elmer ICP Optima 2000DV, with a detection limit between 0.005 and 0.01 ppm, depending upon the element. The quantitative determination of elements was carried out using a calibration curve prepared from Merck’s certified multi-elemental standard solution. It was constructed with eight calibration points in the concentration range from 0 to 8000 ppm.

### 2.3. Tailings Samples

**Figure 1:**
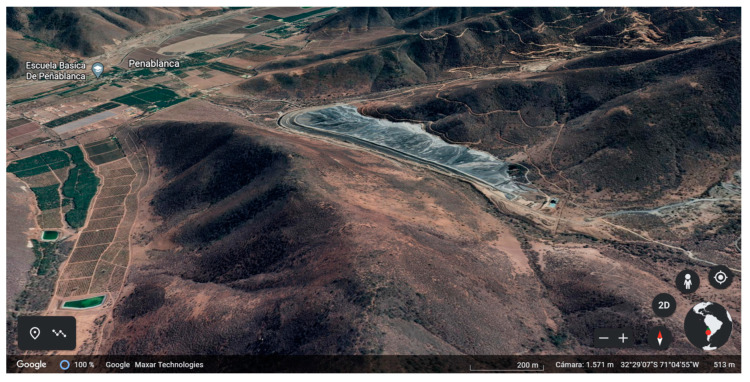
Tailings impoundment, property of Minera Las Cenizas. Google Earth.

The samples were taken from the paste mine tailings impoundment of Minera Las Cenizas, located in Cabildo, in the Region of Valparaíso, whose coordinates are 32°28′16.1″ S, 71°05′00.2″ W. Figure 1 shows a map of the impoundment location.

For the experiments, tailings were ground in a ball mill, sieved under ASTM N°18 (1 mm) mesh, and homogenized. The characterization of tailings, including the determination of pH according to U.S. Environmental Protection Agency Method 9045D (EPA) [37], is presented in Table 1. The content of oxidized compounds was obtained from the published data of Servicio Nacional de Geología y Minería de Chile (Sernageomin) [38].

### 2.4. Evaluation of Phytoremediation

The phytoremediation capability of the plants was evaluated using the translocation factor (TF) and bioconcentration factor (BCF). The former allows determination of the ability of the plant to mobilize the pollutant from roots to shoots (in this case, part of the plant above the ground), while the latter factor indicates the ability to accumulate the target metal in a different part of the plant. TF and BCF are presented by Equations (1) and (2), respectively:

(1) $$\mathrm{TF}=\frac{\mathrm{Metal}\;\mathrm{in}\;\mathrm{aerial}\;\mathrm{part}}{\mathrm{Metal}\;\mathrm{in}\;\mathrm{plant}\;\mathrm{roots}}$$

(2) $$\mathrm{BCF}=\frac{\mathrm{Metal}\;\mathrm{in}\;\mathrm{an}\;\mathrm{especific}\;\mathrm{part}\;\mathrm{of}\;\mathrm{the}\;\mathrm{plant}}{\mathrm{Metal}\;\mathrm{in}\;\mathrm{the}\;\mathrm{tailings}}$$

A value of BCF greater than 1 indicates the potential use of a plant species for phytoremediation. In contrast, a TF > 1 shows the ability for the metal to be translocated from roots to shoots and vice versa; if it is less than 1 (TF < 1), the species is a metal excluder [39,40,41,42,43,44].

Additionally, the removal efficiency (RE%) is used to obtain the percentage of the total mass of the target metal removed from tailings using a plant species according to Equation (3).

(3) $$\mathrm{RE}\%=\frac{C_{i}-C_{f}}{C_{i}}\times 100\%$$

where C_i_ and C_f_ are the initial and final concentrations of the element in the tailings.

### 2.5. Phytoremediation Tests

The phytoremediation experiences were performed ex situ in an outdoor environment at the Chemical and Environmental Engineering Department of Universidad Técnica Federico Santa María, located in Valparaíso (33°02′05” S, 71°35′43″ W). The plants were transplanted into pots with initial heights between 10 and 15 cm, containing 3100 g dry weight of ground and sieved tailings. The experimental and growth period was seven months. Tap water and a liquid foliar organic stimulant based on *Ascophyllum nodosum* were supplied once a week.

At the end of the experimental period, each bush was removed from the pot individually. The plant was carefully washed with tap water, distilled water, and deionized water, and the roots were separated from the stems and leaves. Each part of the plant was cut into small pieces and dried in a laboratory oven at 45 °C until constant weight. Each plant’s stems, leaves, and roots were ground and homogenized separately. In the case of tailings, all roots and plant debris were removed from them, and tailings were dried in a laboratory oven at 105 °C until constant weight and sieved under ASTM N°18 mesh. Representative samples were taken from stems, leaves, and roots by the quartering method.

The digestion procedure was the same for plants and tailings, differing by the amount of sample used, i.e., 0.200 g dry weight in the case of plants and 0.06 g dry weight for tailings. The respective amount of sample was placed in a Teflon vial with 2 mL of 30% hydrogen peroxide and 8 mL of 70% nitric acid. A 4 h pre-digestion stage was carried out before digestion, after which a digestion procedure in a microwave, Ethos Easy, was carried out. Subsequently, the samples were placed in a 25 mL volumetric flask, filled with deionized water, and analyzed by inductively coupled atomic emission spectroscopy (ICP-OES).

### 2.6. Statistical Treatment of the Data

Five bushes of each species were analyzed, taking three samples for each part of the plant and three samples of tailings, and the value of concentration was the mean of three measurements. First, to compare the metal concentration in the roots or aerial parts, a one-way analysis of variance (two-way ANOVA) was performed with a significance level of *p* < 0.05 to compare means. Tukey’s test was carried out to compare the different species.

## 3. Results

### 3.1. Blank Samples and Initial Concentrations

Three blank samples of each species were used. Their initial and final concentrations of Cr, Ni, and Zn in aerial parts and roots are presented in Table 2 as mean concentration and standard deviation.

**Figure 2:**
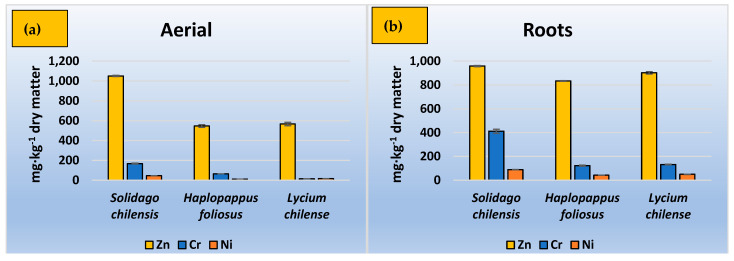
The initial and final concentration of heavy metals. (**a**) Aerial parts, (**b**) roots.

According to Figure 2, *Solidago chilensis* presented higher concentrations among the species studied in roots and aerial parts for all metals—Zn, Cr and Ni. In the case of Zn, the concentration in the aerial parts slightly exceeded its concentration in roots, at around 1000 mg·kg^−1^. *Haplopappus foliosus* and *Lycium chilense* presented concentrations of Zn of 834 and 902 mg·kg^−1^ in roots, respectively, and approximately 550 mg·kg^−1^ in aerial parts in both cases.

Chromium was accumulated to a much greater extent in roots than in aerial parts for all species studied; for *Haplopappus foliosus* and *Lycium chilense,* the concentration of total Cr in roots was close to 130 mg·kg^−1^ in both cases, while the concentration in the roots in the case of *Solidago chilensis* was 411 mg·kg^−1^. For the aerial parts, the concentration in *Haplopappus foliosus* and *Solidago chilensis* was about half that found in the roots, and *Lycium chilense* was much lower.

### 3.2. TF, BCF, and Removal Efficiency

**Figure 3:**
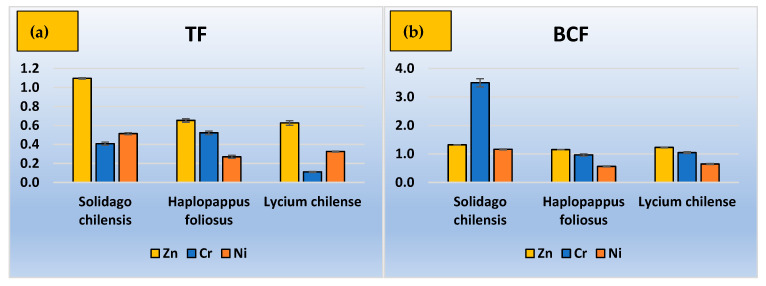
(**a**) Translocation factor, (**b**) bioconcentration factor.

The translocation and bioconcentration factors were calculated for each plant and metal, and the mean values are presented in Figure 3. As can be seen from this figure, almost all species showed a TF < 1, except *Solidago chilensis*, which exhibited TF = 1.1 for Zn. The same species presented a value of BCF higher than 1 for all elements under study, with an extremely high value of 3.5 for Cr; therefore, *Solidago chilensis* could be a candidate for Zn phytoextraction, and a BCF > 1 with a TF < 1 indicates a tendency towards the phytostabilization of Cr and Ni.

In the case of *Haplopappus foliosus* and *Lycium chilense*, the translocation factors for all metals studied were lower than 1. Both species showed a bioconcentration factor slightly greater than 1 for Zn and close to it for Cr.

**Figure 4:**
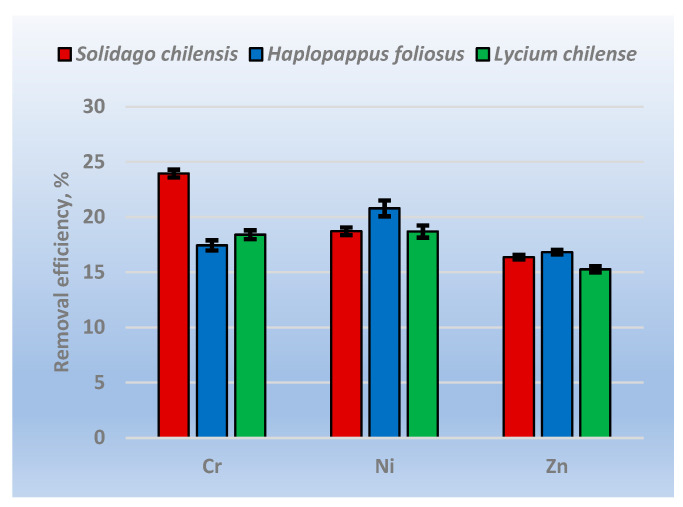
Removal efficiency.

As can be seen from Figure 4, the maximum removal efficiency of 24% was obtained for Cr with *Solidago chilensis*, and the maximum removal efficiency for Ni and Zn was obtained with *Haplopappus foliosus* was 20.8% and 16.8%, respectively. Among the three metals analyzed, Zn presented the lower removal efficiency values, between 15 and 17%; the removal efficiency for Ni was in the range of 18.5% and 21%, and finally, for Cr, values between 17% and 24% were obtained.

Considering the factors for phytoremediation assessment and removal efficiency, *Solidago chilensis* seems to be the most promising species among the species analyzed.

## 4. Discussion

To try to overcome the disadvantages associated with the application of a phytoremediation process, native or endemic species were chosen to realize this study; these types of plants present better adaptation to climatic conditions and the biological environment [45], and rapid growth and significant biomass yield was also desired [46].

*Solidago chilensis*, *Haplopappus foliosus,* and *Lycium chilense* were the chosen species, and no previous studies of Zn, Cr, and Ni phytoremediation using these species were found. It is essential to mention that concentrations of As and Hg were below the detection limit in the roots and aerial parts. Cu concentrations for *Solidago chilensis* were reported in previous research [47].

Since there are no Chilean regulations limiting the concentration of heavy metals in soils, the Dutch Soil Quality Standards will be used for comparison (See Table 3) [48,49]. In this case, the concentration of Zn and Cu in tailings surpasses the intervention value. Ni exceeds the target value, and its concentration is very close to the intervention value, corresponding to 95% of its value. In the case of Cr, all used concentrations in this paper and the standard presented in Table 3 correspond to the total chromium; its value exceeds the target value of 100 mg·kg^−1^ established by the Dutch standard.

In the case of *Solidago chilensis*, the concentration of Zn in roots and aerial parts was around 1000 mg·kg^−1^ dry weight. Although high, this value does not classify it as a hyperaccumulator. The values BCF = 1.3 and TF = 1.1 demonstrate the potential ability of this species to stabilize Zn, with little ability to extract it.

In the study of Lam et al., 2017 [34], translocation for Zn was close to 1.5 with the native tree *Prosopis Tamarugo* in a substrate of tailings without amendments.

In another study, *Schninus polygama* (Huingán), *Baccharis, linearis* (Romero), *Trevoa quinquenervia* (Tralhuén), and *Muehlenbeckia hastulata* (Quilo), all native Chilean evergreen shrubs, were tested in situ, obtaining, for Tralhuén and Romero, values of TF equal to 2.3 and 4.2, respectively. In addition, the species *Prosopis chilensis* (Algarrobo), *Acacia caven* (Espino), and *Quillaja saponaria* (Quillay), all trees, also presented a TF higher than 1 [35].

The same study reports a concentration of 50 mg·kg^−1^ for Zn in the leaves of Romero and 13 mg·kg^−1^ in the roots of Quillay as the maximum detected concentrations.

The exact metals analyzed in a previous study [50] using *Oxalis gigantea*, *Cistanthe grandiflora,* and *Puya beteroniana* presented for the last two species BCF values of 1.4 and 1.7, which are similar results to those for Zn and Ni obtained here. However, the removal efficiencies were lower, achieving a maximum of 10% for Cr, 15% for Ni, and 6.5% for Zn. In the specific case of Cr, no endemic Chilean species have been cataloged as hyperaccumulators [50]; only a few hyperaccumulator species have been found, and most grow under tropical climate conditions [51,52]. Some species recognized as useful for the phytoremediation of Cr are *Plunchea indica*, *Cynodon dactylon*, *Phragmites australis*, *Typha angustifolia*, *Pterocarpus indicus*, and *Jatropha curcas* [52]. In this study, *Solidago chilensis* accumulated a concentration greater than 400 mg·kg^−1^ dry weight of Cr, with a clear tendency to stabilize it, and a removal efficiency of 24% in seven months. This plant grows throughout almost the entire Chilean territory, and is very frequently occurring, which is an advantage when wanting to implement a phytoremediation process with it. In the study of Milla-Moreno et al., 2021, reported values of Cr were under 2 mg·kg^−1^ in an on-site phytoremediation experiment for the species Espino, Quillay, Romero, and Tralhuén in leaves and roots [35].

It has been well established that chromium (III) can be oxidized to chromium (VI) in the presence of organic matter, oxygen, manganese dioxide, moisture, and high temperatures [53,54,55]; therefore, although the speciation of chromium was not performed in this study, the occurrence of both species is highly probable. As can be seen from the results, the chromium concentration in the roots far exceeds the concentration in the aerial parts, as was indicated by Calder et al., 1988 and Stackhouse and Benson 1989 [56,57], establishing the low mobility of chromium for translocation from roots to shoots. None of the species under study presented the ability to translocate Cr, but *Haplopappus foliosus* and *Lycium chilense* showed a bioconcentration factor of a little more than 1, while *Solidago chilensis* was close to 3.5; therefore, these species would be potential candidates to phytostabilize this metal. Further work is needed to speciate chromium in tailing and plant parts.

With respect to toxicity, all species surpassed the threshold for Cr of 5 mg·kg^−1^, where the lower detected concentration was 14.5 mg·kg^−1^ for aerial parts and *Lycium chilense* and the higher concentration equal to 411.5 mg·kg^−1^ was obtained for roots and *Solidago chilensis*. In the case of Zn, toxicity is considered to be present at concentrations higher than 300 mg·kg^−1^, which is the case of all species in roots and aerial parts, and the higher concentration for Zn was achieved with *Solidago chilensis* for all parts of the plants with values over or very close to 1000 mg·kg^−1^. Finally, the toxicity threshold for Ni was mostly surpassed in roots, and the maximum concentration of 82.2 mg·kg^−1^ was detected in *Solidago chilensis*.

The most notable results were obtained for Cr and Zn with *Solidago chilensis,* and no remarkable results were obtained in the case of Ni.

## 5. Conclusions

In this study, three species that naturally grow in the Northern and Central zone of Chile were used, and their abilities to remove Cr, Ni and Zn were tested; these species have not been previously studied for the phytoremediation of these metals and were chosen due to their high resistance and low water requirements. The experiments were carried out over seven months in outdoor conditions, and the translocation and bioconcentration factors were obtained together with the removal efficiency. The best performance for Cr was achieved with *Solidago chilensis*, with a removal efficiency of 24%. This species shows an apparent ability to phytostabilize this element with a bioconcentration factor of 3.5 and was the only one with a translocation factor for Zn slightly greater than 1. *Happlopappus foliosus* and *Licyum chilense* showed a BCF for Zn and Cr close to 1, and a removal efficiency close to 15% was obtained with all species for Zn. The highest removal efficiency for Ni (20%) was obtained with *Haplopappus foliosus*. No outstanding values of TF and BCF were obtained for Ni. Related to the toxicity concentration in plants, *Solidago chilensis* was the most resistant species for Zn, Cr, and Ni, with detected concentrations well above the threshold level, mainly in the case of Cr.

## Figures and Tables

**Table 1:** Characterization of tailing sample.

Parameter	Value	Units
Specific gravity	2.78 ± 0.25	-
pH	7.30 ± 0.10	-
Solid concentration in weight	82.00 ± 1.00	%
Granulometry d_50_	0.046 ± 0.001	µm
As	90 ± 4.53	mg·kg^−1^ dry tailing
Hg	Under detection limit	mg·kg^−1^ dry tailing
Cu	1582.22 ± 78.31	mg·kg^−1^ dry tailing
Pb	228.15 ± 2.79	mg·kg^−1^ dry tailing
Zn	869.80 ± 31.54	mg·kg^−1^ dry tailing
Ni	94.64 ± 2.57	mg·kg^−1^ dry tailing
Mo	3.86 ± 0.17	mg·kg^−1^ dry tailing
Cd	Under detection limit	-
Cr	154.63 ± 5.41	mg·kg^−1^ dry tailing

**Table 2:** Blank samples. Initial and final concentration.

Element	Concentration mg·kg^−1^ Dry Weight ± Standard Deviation		
	*Solidago chilensis*	*Haplopappus foliosus*	*Lycium chilense*
Cr aerial part t = 0	<d.l.	<d.l.	<d.l.
Cr aerial part t = 7 months	<d.l.	<d.l.	<d.l.
Cr roots t = 0	0.01 ± 0.00	0.01 ± 0.00	<d.l.
Cr roots t = 7 months	0.01 ± 0.00	0.01 ± 0.00	<d.l.
Ni aerial part t = 0	3.00 ± 0.11	4.59 ± 0.02	5.03 ± 0.02
Ni aerial part t = 7 months	3.25 ± 0.09	5.01 ± 0.04	5.17 ± 0.17
Ni roots t = 0	3.58 ± 0.13	5.53 ± 0.14	6.46 ± 0.13
Ni roots t = 7 months	3.67 ± 0.02	6.23 ± 0.18	6.59 ± 0.10
Zn aerial part t = 0	15.04 ± 0.11	14.32 ± 0.22	15.11 ± 0.09
Zn aerial part t = seven months	15.15 ± 0.09	15.45 ± 0.00	15.33 ± 0.19
Zn roots t = 0	16.45 ± 0.28	18.01 ± 0.22	15.98 ± 0.08
Zn roots t = 7 months	17.05 ± 0.20	18.11 ± 0.28	16.07 ± 0.12

**Table 3:** Target values for soil with 10% organic matter and 25% clay.

Parameter	Target Value	Maximum Value for Residential Land Use	Intervention Value	Units
Zn	140	200	720	mg·kg^−1^ dry sediment
Ni	35	39	100	mg·kg^−1^ dry sediment
Cr	100	-	380	mg·kg^−1^ dry sediment

## Data Availability

Not applicable.
